# Seronegative Autoimmune Encephalitis Presenting With Severe Neuropsychiatric Symptoms in an Older Adult: A Case of Diagnostic Complexity

**DOI:** 10.1155/crps/7669338

**Published:** 2026-06-25

**Authors:** Christopher E. Potts, Akbar Ali, James Issa, Usman Alizai, Dakota May

**Affiliations:** ^1^ Marshall University Joan C. Edwards School of Medicine, Huntington, West Virginia, USA, marshall.edu; ^2^ The Ohio State University Wexner Medical Center, Columbus, Ohio, USA, osu.edu

**Keywords:** autoimmune encephalitis (AE), autoimmune psychosis, case report, corticosteroid therapy, inflammation, neuroimmunology, neuropsychiatric symptoms, seronegative

## Abstract

Autoimmune encephalitis (AE) represents a potentially reversible cause of subacute cognitive and psychiatric decline. When no autoantibodies are detected, seronegative AE remains diagnostically challenging, particularly among older adults presenting with new‐onset neuropsychiatric symptoms. This report describes an older adult with recurrent episodes of confusion, aphasia, and psychosis whose diagnostic studies—including magnetic resonance imaging (MRI) and cerebrospinal fluid (CSF) analysis—were unremarkable. The individual had previously demonstrated substantial improvement after corticosteroid therapy and was again treated empirically with immunotherapy following psychiatric consultation. This case highlights the importance of maintaining a high index of suspicion for AE when standard serologic markers are absent and underscores the collaborative role of psychiatry and neurology in evaluating rapidly progressive cognitive and behavioral syndromes, which may present initially as primary psychiatric illness. Early consideration of autoimmune etiologies and prompt initiation of immunotherapy can significantly improve outcomes in patients with otherwise unexplained, rapidly progressive cognitive or behavioral syndromes.

## 1. Introduction

Autoimmune encephalitis (AE) is characterized by the subacute onset of immune‐mediated neuropsychiatric and cognitive symptoms. Over the past two decades, the discovery of pathogenic autoantibodies has transformed the diagnostic and therapeutic landscape of AE, establishing it as a major cause of rapidly progressive encephalopathy that is both treatable and potentially reversible [1]. However, strong clinical reliance on serologic confirmation has led to the recognition of seronegative AE subtypes that lack a specific biomarker yet share similar clinical and immunopathologic features with antibody‐positive subtypes. In such cases, accurate diagnosis and clinical improvement depend on clinical suspicion, evidence of inflammation, and the exclusion of alternate causation.

In a popular paper by Graus et al. [1], diagnostic criteria for possible and probable seronegative AE were established, emphasizing that autoantibody serologic detection should not delay diagnosis or initiation of therapy. The criteria acknowledge that many patients present with normal or subclinical magnetic resonance imaging (MRI) or cerebrospinal fluid (CSF) findings and that psychiatric manifestations—including psychosis, affective instability, and catatonia—can dominate the clinical picture early in its course [1]. Recognition of these atypical neuropsychiatric presentations has led to a paradigm shift, with psychiatry now playing an essential role in the early detection of seronegative AE, particularly in cases initially attributed to primary psychiatric illness.

Psychiatric symptoms are among the most common initial features of all subtypes of AE, often preceding significant neurological findings by days to weeks. In a recent multicenter cohort study of 642 patients by Bean et al. [2], more than half of patients presenting with autoimmune or infectious encephalitis first presented to psychiatric services before other subspecialties, highlighting the importance of psychiatric involvement in timely diagnosis and treatment. Similarly, a study by Tebartz van Elst et al. [3] introduced the idea of *autoimmune psychosis*, describing the clinical overlap between AE and primary psychiatric disorders and promoting systematic immunologic screening. Atypical phenotypes of primary psychiatric illness warranting a neurology consult include rapid onset, fluctuating course, abnormal movements, poor response to psychotropics, and prior steroid responsiveness.

This report describes a 68‐year‐old male with no prior history of psychiatric illness who developed recurrent episodes of subacute neuropsychiatric decline and behavioral disturbance and ultimately was diagnosed with possible autoantibody‐negative AE. By documenting recurrent steroid‐responsive encephalopathy in an older adult lacking serologic, neuroimaging, and CSF abnormalities, this report illustrates the diagnostic value of prior treatment response as a clinical anchor when standard biomarkers are uninformative and highlights psychiatry’s role in timely recognition and treatment.

## 2. Case Description

A 68‐year‐old male with no prior psychiatric history presented to the emergency department with 3 weeks of progressive confusion, emotional instability, and disorganized behavior. Family members reported that the abrupt onset of symptoms and cognitive decline was significant in pressured and tangential speech, visual hallucinations, and insomnia. The patient was increasingly irritable, displaying atypical symptoms of inappropriate laughter and purposeless limb movement abnormalities. There was no prior history of substance use, recent medication adjustment, or infection reported.

On arrival, the patient was alert and oriented only to person and place. Speech was fragmented and occasionally incoherent, with incidences of echolalia and word salad. The patient was labile in affect, frequently and inconsistently alternating between irritability and laughter. Neurology was consulted, and the examination revealed intermittent orofacial dyskinesia, mild rigidity in the upper extremities, and fine myoclonic movements without focal weakness or sensory loss. There was no evidence of autonomic abnormalities, and the vital signs remained stable. Routine laboratory evaluation—including CBC, CMP, TSH, B12, and infection serology panels—was within normal limits. Urine and serum toxicology screens were negative. A noncontrast head CT and brain MRI revealed no acute pathology apart from mild chronic microvascular changes (Figure 1).

**Figure 1:**
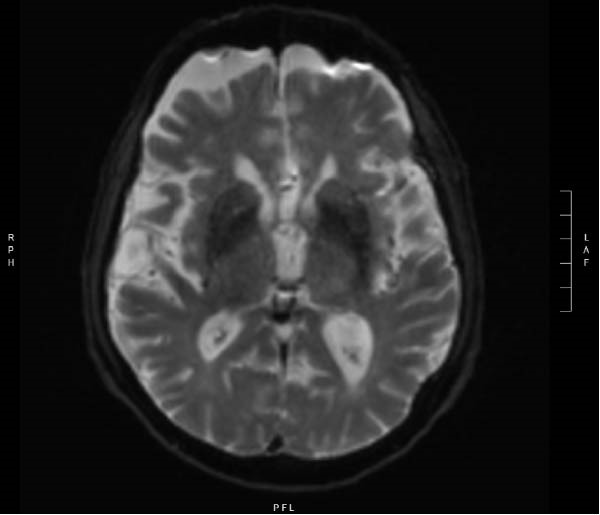
Axial T2‐weighted brain MRI showing mild chronic microvascular white‐matter changes without abnormal enhancement, edema, or focal lesions. No acute pathology observed.

CSF obtained by lumbar puncture was clear and acellular with normal WBC counts, RBC counts, protein, and glucose (Figure 2). Bacterial cultures, viral cultures, and PCR testing for HSV‐1/1, VZV, CMV, and enterovirus were negative. Autoimmune and paraneoplastic panels for both serum and CSF—including NMDA receptor antibodies, LGI1, CASPR2, AMPA, and GABA‐B receptors—were unremarkable. Serum thyroid peroxidase, thyroglobulin, ANA, and ANCA were also negative. A whole body set of CT scans did not result in the detection of any malignancies. The electroencephalogram demonstrated diffuse slowing without epileptiform discharges, consistent with mild encephalopathic changes.

**Figure 2:**
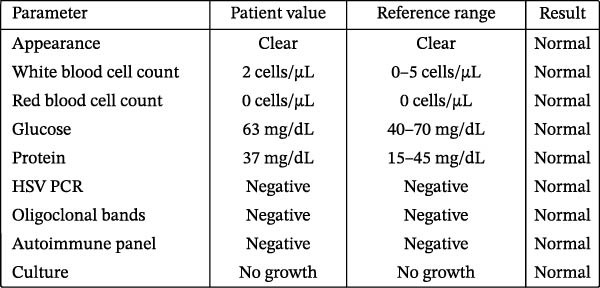
Cerebrospinal fluid (CSF) analysis demonstrating normal parameters, including cell counts, glucose, and protein, with negative results for HSV PCR, oligoclonal bands, autoimmune panels, and infectious panels.

The patient was admitted for further evaluation. Psychiatry was consulted for management of agitation and behavioral disturbances. Risperidone was initiated but resulted in no significant clinical benefit. Over the course of the following days, the patient experienced fluctuation in attention, increased speech latency, perseveration, occasional mutism, and overall neurocognitive decline. During this time, abnormal face movements persisted. A thorough review of medical records revealed a similar chain of events that presented 2 years earlier. The event was nearly identical and consistent with confusion, paranoia, and behavioral disinhibition. Similarly, all serologic workup again ruled out infectious or autoimmune causation. All symptoms in this previous event resolved completely following a course of high‐dose corticosteroids and the patient was discharged following symptomatic improvement.

On current presentation, empiric therapy for possible autoantibody‐negative AE was initiated due to the subacute progression, lack of strictly identifiable etiology, and history of prior steroid responsiveness with a similar presentation. Intravenous methylprednisolone (1 g) daily was administered for a 5‐day course and followed by an oral taper. Over the next week, steady symptomatic improvement was described, including coherent speech, normalized sleep, and stabilized affect. By the end of the treatment course, the patient was fully oriented with no residual symptoms (Figure 3).

**Figure 3:**
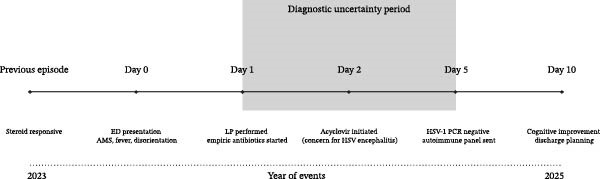
Clinical timeline of events. Initial ED presentation with fever, altered mental status, and disorientation (Day 0). Marked symptomatic improvement was observed by Day 10. The shaded region represents the highlighted diagnostic uncertainty period.

The patient was discharged to an inpatient rehabilitation center and later followed up with neurology and psychiatry. At 1‐month follow‐up, the patient was stable and regained the function of daily living activities with only mild residual short‐term memory loss. The patient was referred to a tertiary neuroimaging center for long‐term monitoring. The recurrence of steroid‐responsive encephalopathic syndrome in the absence of identifiable serologic evidence supported a final diagnosis of possible autoantibody‐negative AE.

## 3. Discussion

Seronegative AE remains one of the most diagnostically challenging entities in neuroimmunology. Despite meeting clinical criteria, a significant number of patients lack confirmatory serologic evidence. Thus, AE is a clinical diagnosis that is supported by—yet not defined by—serologic results [4].

In this case, the patient did not strictly meet criteria for probable autoantibody‐negative AE as defined by Graus et al. [1] because MRI and CSF analysis showed no abnormalities supportive of AE, and brain biopsy was not pursued. The presentation is therefore best classified as possible AE, while still demonstrating strong clinical features supporting an autoimmune etiology, including subacute onset, prominent neuropsychiatric and movement features, exclusion of alternative causes, and—most notably—a prior near‐identical episode that responded fully to corticosteroids. Recent studies emphasize that the spectrum of seronegative AE is much broader than previously appreciated and likely reflects diverse immune mechanisms and clinical phenotypes. Large‐scale analyses have shown that nearly 60% of clinically suspected AE cases lack detectable antibodies, highlighting that the absence of serology does not exclude autoimmune pathology [4].

Emerging research has sought to enhance predictive measures for patients with suspected AE. The Girona (ACPE‐Gi) score developed in 2025 is an integrated tool that works to assess acute severity and forecast disability risk by incorporating mental status changes, autonomic dysfunction, and seizure burden [5]. Similarly, a Frontiers in Neurology study by Xie et al. [6] proposed that a predictive measure using clinical and laboratory variables such as psychiatric disturbance, cognitive impairment, and autonomic instability may be valuable in identifying patients at risk for severe AE. Together, these findings support that mental status and autonomic instability carry prognostic significance for AE and are commonly initially evaluated by psychiatry services.

Neuropsychiatric presentations continue to dominate the early course of seronegative AE cases, particularly resulting in frequent diagnostic delay. Case reports and meta‐analyses demonstrate that abnormal movements, particularly orofacial dyskinesia and dystonia, together with psychiatric abnormalities can serve as valuable diagnostic clues [7, 8]. In this case, orofacial dyskinesia and psychiatric involvement paralleled cognitive decline, and recognition of these features by psychiatric services was crucial in prompting immunotherapy and specialist referral.

The pathophysiology of seronegative AE is an immune‐mediated cascade of events that is heterogeneous by nature and warrants individualized workup and evaluation. In addition to the well‐understood autoantibody and T‐cell‐mediated inflammation seen in these patients, recent literature suggests that transplant recipients being treated with calcineurin inhibitors—such as cyclosporine—may be at higher risk for AE [9]. This rare case highlights the importance of individualized workup for patients with suspected AE due to the immunologic heterogeneity underlying the pathophysiological mechanism of the disease.

Treatment outcomes for patients with all subtypes of AE are dependent on timely intervention. Early initiation of corticosteroids, intravenous immunoglobulin (IVIG), or plasmapheresis is associated with better recovery regardless of serologic status [10]. Patients receiving delayed therapy—often due to diagnostic uncertainty or a relapsing‐remitting course—have higher modified Rankin Scale scores and a higher likelihood of poor cognitive recovery [10]. When patients fail to respond to first‐line immunotherapy, second‐line agents such as rituximab and cyclophosphamide are commonly employed, and longer‐term immunosuppression with mycophenolate mofetil or azathioprine may be considered for relapsing diseases. Alongside disease‐modifying therapy, symptomatic management is essential. Antipsychotics may be used for psychosis and agitation, though clinicians should monitor closely for extrapyramidal effects and neuroleptic malignant syndrome–like reactions, which appear to occur with greater frequency in AE. Benzodiazepines are first‐line for catatonia, and antiepileptic drugs are used for seizures. Electroconvulsive therapy (ECT) has emerged as a valuable adjunctive treatment for severe psychiatric manifestations of AE, particularly catatonia, treatment‐refractory psychosis, and severe agitation that is unresponsive to pharmacotherapy and may be administered concurrently with immunotherapy [11]. The favorable symptomatic and clinical resolution in this case following corticosteroid intervention reinforces the importance of timely empiric immunotherapy when AE is clinically suspected, even in the absence of serologic confirmation.

This case highlights the evolving interface between psychiatry and neurology services. In cases of suspected AE, early collaboration may be decisive in preventing irreversible decline and is the most effective strategy for optimizing outcomes in atypical cases of seronegative AE.

## 4. Conclusion

This case contributes to the growing body of literature on seronegative AE in three specific ways. First, it documents recurrent steroid‐responsive encephalopathy in an older adult—a demographic underrepresented in existing AE cohorts, where attention has more often centered on younger patients with anti‐NMDAR encephalitis. Second, it illustrates how a prior episode of corticosteroid‐responsive neuropsychiatric decline can serve as one of the most informative diagnostic anchors when contemporaneous biomarkers (MRI, CSF, and serology) are unrevealing—a clinical pattern that warrants explicit consideration in the workup of recurrent unexplained encephalopathy. Third, it reinforces that psychiatric clinicians, who are often the first to encounter such patients, are positioned to recognize the atypical features (subacute onset, abnormal movements, fluctuating course, poor antipsychotic response, and prior steroid responsiveness) that should prompt early neuroimmunology referral and empiric immunotherapy when standard diagnostic testing is inconclusive. We hope this report adds practical diagnostic anchors for clinicians evaluating older adults with otherwise unexplained subacute neuropsychiatric decline and supports the therapeutic value of early empiric intervention even in the absence of serologic confirmation.

## Author Contributions

**Christopher E. Potts**: writing – original draft, writing – review and editing, conceptualization, investigation. **Akbar Ali, James Issa, and Usman Alizai**: writing – review and editing, conceptualization, investigation. **Dakota May**: project administration, supervision, writing – review and editing, conceptualization.

## Funding

The authors received no specific grant or financial support from any funding agency in the public, commercial, or not‐for‐profit sectors for the preparation of this case report.

## Disclosure

An earlier version of this work was presented as a poster at the Association of Medicine and Psychiatry (AMP) 2025 Annual Conference, held on October 9, 2025, in Newport Beach, California.

## Ethics Statement

This single‐patient case report was IRB exempt under institutional policy, under the Marshall University Medical IRB.

## Consent

Written informed consent was obtained from the patient’s next of kin for the publication of this case report and accompanying images. All identifying patient information has been removed to protect patient privacy. The report was prepared in accordance with institutional ethical standards and the CARE guidelines.

## Conflicts of Interest

The authors declare no conflicts of interest.

## Supporting Information

Additional supporting information can be found online in the Supporting Information section.

## Supporting information


**Supporting Information** Completed CARE checklist submitted in accordance with CARE reporting guidelines.

## Data Availability

All data supporting the conclusions of this article are included within the article.

## References

[1] Graus F. , Titulaer M. J. , and Balu R. , et al.A Clinical Approach to Diagnosis of Autoimmune Encephalitis, The Lancet Neurology. (2016) 15, no. 4, 391–404, 10.1016/S1474-4422(15)00401-9. 26906964 PMC5066574

[2] Bean P. , Heck A. , and Habis R. , et al.Psychiatric Manifestations of Encephalitis, Annals of Clinical and Translational Neurology. (2025) 12, no. 2, 405–414, 10.1002/acn3.52260. 39776337 PMC11822797

[3] Tebartz van Elst L. , Runge K. , Meyer P. T. , Urbach H. , Venhoff N. , and Prüss H. , The Neuropsychiatric Checklist for Autoimmune Psychosis: A Narrative Review, Biological Psychiatry. (2025) 98, no. 9, 654–669, 10.1016/j.biopsych.2025.02.889. 39987981

[4] van Steenhoven R. W. and Titulaer M. J. , Seronegative Autoimmune Encephalitis: Exploring the Unknown, Brain. (2022) 145, no. 10, 3339–3340, 10.1093/brain/awac338. 36111366 PMC9586533

[5] Alvarez Bravo G. , Guglielmini G. , and Quiroga Varela A. , et al.Assessment of Clinical Prognosis in Autoimmune Encephalitis: Girona Score, Frontiers in Psychiatry. (2025) 16, 10.3389/fpsyt.2025.1447009, 1447009. 39944213 PMC11814168

[6] Xie Z. , Zhang J. , Liu L. , Hu E. , and Wang J. , Prediction Model for Severe Autoimmune Encephalitis: A Tool for Risk Assessment and Individualized Treatment Guidance, Frontiers in Neurology. (2025) 16, 10.3389/fneur.2025.1575835, 1575835. 40170898 PMC11958171

[7] Siriratnam P. , McArthur L. , Chen Z. , Kempster P. , and Monif M. , Movement Disorders in Cell Surface Antibody Mediated Autoimmune Encephalitis: A Meta-Analysis, Frontiers in Neurology. (2023) 14, 10.3389/fneur.2023.1225523, 1225523. 37545714 PMC10401600

[8] Li H. , Chen J. , Zhou P. , and Meng Q. , Analysis of Characteristics of Movement Disorders in Patients With Anti-N-Methyl-D-Aspartate Receptor Encephalitis, Frontiers in Neurology. (2024) 15, 10.3389/fneur.2024.1357697, 1357697. 38497042 PMC10941647

[9] Holzl K. and Albasini F. , Possible Tacrolimus-Associated Seronegative Autoimmune Encephalitis Presenting With Subacute Cognitive Impairment and Disorientation - Case Report, Frontiers in Medicine. (2025) 12, 10.3389/fmed.2025.1540960, 1540960. 40432728 PMC12106454

[10] Titulaer M. J. , McCracken L. , and Gabilondo I. , et al.Treatment and Prognostic Factors for Long-Term Outcome in Patients With Anti-NMDA Receptor Encephalitis: An Observational Cohort Study, The Lancet Neurology. (2013) 12, no. 2, 157–165, 10.1016/S1474-4422(12)70310-1. 23290630 PMC3563251

[11] Warren N. , Grote V. , O’Gorman C. , and Siskind D. , Electroconvulsive Therapy for Anti-N-Methyl-d-Aspartate (NMDA) Receptor Encephalitis: A Systematic Review of Cases, Brain Stimulation. (2019) 12, no. 2, 329–334, 10.1016/j.brs.2018.11.016. 30528383

