# Supplementary material for: Seronegative Autoimmune Encephalitis Presenting With Severe Neuropsychiatric Symptoms in an Older Adult: A Case of Diagnostic Complexity
Source: Case Rep Psychiatry. 2026 Jun 25;2026:7669338. doi: 10.1155/crps/7669338 (PMC13305320; doi:10.1155/crps/7669338)
Supplement: Supplementary file 1 — Supporting Information Completed CARE checklist submitted in accordance with CARE reporting guidelines. [file CRPS-2026-7669338-s001.pdf]

CARE
case report guidelines

CARE Checklist of information to include when writing a case report

(cc) BY-NC-ND

Topic	Item	Checklist item description	Reported on Line
Title	1	The diagnosis or intervention of primary focus followed by the words "case report"	1-2
Key Words	2	2 to 5 key words that identify diagnoses or interventions in this case report, including "case report"	29-31
Abstract (no references)	3a	Introduction: What is unique about this case and what does it add to the scientific literature?	14-27
	3b	Main symptoms and/or important clinical findings	17-21
	3c	The main diagnoses, therapeutic interventions, and outcomes	20-26
	3d	Conclusion—What is the main "take-away" lesson(s) from this case?	24-26
Introduction	4	One or two paragraphs summarizing why this case is unique (**may include references**)	33-63
Patient Information	5a	De-identified patient specific information.	65-67
	5b	Primary concerns and symptoms of the patient.	66-70
	5c	Medical, family, and psycho-social history including relevant genetic information	70-71, 96-99
	5d	Relevant past interventions with outcomes	96-100
Clinical Findings	6	Describe significant physical examination (PE) and important clinical findings.	72-77
Timeline	7	Historical and current information from this episode of care organized as a timeline	223-226
Diagnostic Assessment	8a	Diagnostic testing (such as PE, laboratory testing, imaging, surveys).	78-90
	8b	Diagnostic challenges (such as access to testing, financial, or cultural)	83-87, 115-142
	8c	Diagnosis (including other diagnoses considered)	111-113
	8d	Prognosis (such as staging in oncology) where applicable	109-113, 163-168
Therapeutic Intervention	9a	Types of therapeutic intervention (such as pharmacologic, surgical, preventive, self-care)	102-106
	9b	Administration of therapeutic intervention (such as dosage, strength, duration)	103-104
	9c	Changes in therapeutic intervention (with rationale)	92-99
Follow-up and Outcomes	10a	Clinician and patient-assessed outcomes (if available)	109-113
	10b	Important follow-up diagnostic and other test results	110-111
	10c	Intervention adherence and tolerability (How was this assessed?)	104-107
	10d	Adverse and unanticipated events	104-107
Discussion	11a	A scientific discussion of the strengths AND limitations associated with this case report	115-161
	11b	Discussion of the relevant medical literature **with references**.	193-213
	11c	The scientific rationale for any conclusions (including assessment of possible causes)	152-157, 163-168
	11d	The primary "take-away" lessons of this case report (without references) in a one paragraph conclusion	163-168
Patient Perspective	12	The patient should share their perspective in one to two paragraphs on the treatment(s) they received	n/a
Informed Consent	13	Did the patient give informed consent? Please provide if requested next of kin	Yes ☑ No ☐
